# Pituitary Adenoma and Hyperprolactinemia Accompanied by Idiopathic Granulomatous Mastitis

**DOI:** 10.1155/2017/3974291

**Published:** 2017-02-22

**Authors:** Sebahattin Destek, Vahit Onur Gul, Serkan Ahioglu, Kursat Rahmi Serin

**Affiliations:** ^1^Department of General Surgery, Bezmialem Vakıf University School of Medicine, Istanbul, Turkey; ^2^General Surgery Department, Edremit Government Hospital, Edremit, 10300 Balikesir, Turkey; ^3^Biochemistry Department, Edremit State Hospital, Edremit, 10300 Balikesir, Turkey; ^4^General Surgery Department, Liv Hospital, Ulus, Istanbul, Turkey

## Abstract

Idiopathic granulomatous mastitis (IGM) is a rare chronic inflammatory disease of the breast, and its etiology remains not fully elucidated. IGM is observed more often in patients with autoimmune disease. Hyperprolactinemia is observed during pregnancy, lactation, and a history of oral contraceptive use. A 39-year-old patient with no history of oral contraceptive use presented with complaints such as redness, pain, and swelling in her left breast. Ultrasound and magnetic resonance imaging (MRI) revealed a suspicious inflamed mass lesion. Core biopsy was performed to exclude breast cancer and to further diagnose. The breast abscess was drained and steroids were given for treatment. In order to monitor any progression during the three months of treatment, hormone levels were routinely examined. Prolactin level was above the reference range, and pituitary MRI revealed a pituitary prolactinoma. After treatment with prolactin inhibitors, IGM also improved with hyperprolactinemia. This report emphasizes attention to hyperprolactinemia in cases of IGM diagnosis and treatment.

## 1. Introduction

IGM is a recurrent chronic inflammatory disease characterized by noncaseating granuloma, lobule inflammation, and rare breast abscess formation. Clinical and radiological features may be indistinguishable from breast cancer [1]. The etiology is not fully elucidated [2]. IGM is observed more often in patients with autoimmune diseases, hyperprolactinemia conditions such as pituitary adenomas, during pregnancy and/or lactation, and a history of oral contraceptive use [2, 3]. In the absence of pregnancy and/or lactation, with no history of oral contraceptive use, and/or any additional illness, it is necessary to evaluate prolactin levels during the process of analyzing IGM etiology. If positive, high prolactin levels should be treated primarily.

## 2. Case Report

**Figure 1:**
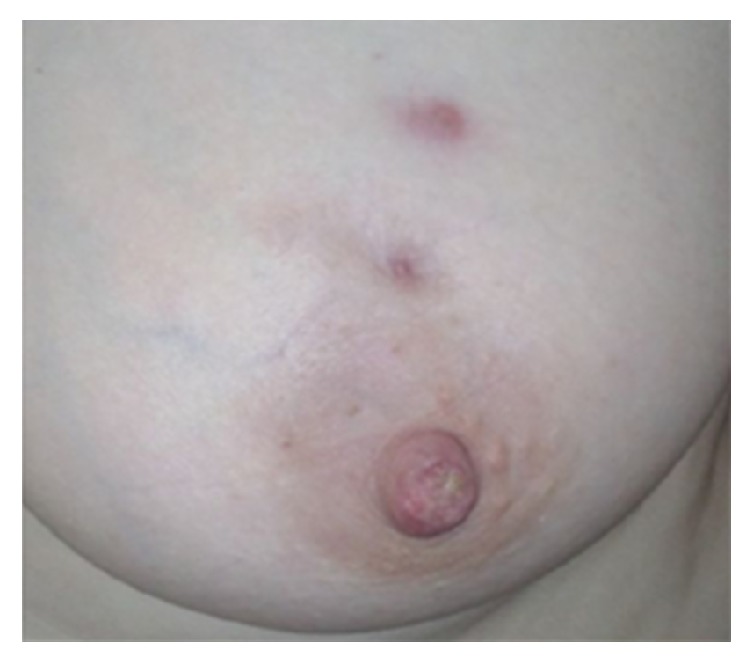
Left breast IGM 1.

**Figure 2:**
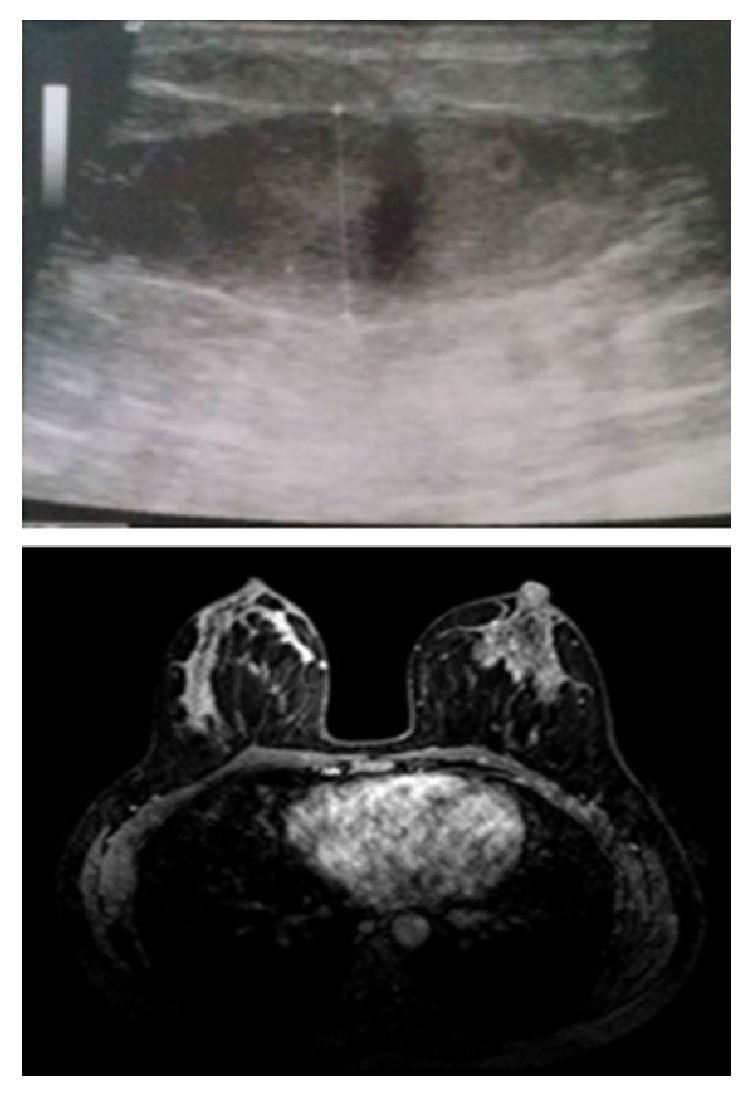
IGM breast USG and MRI.

**Figure 3:**
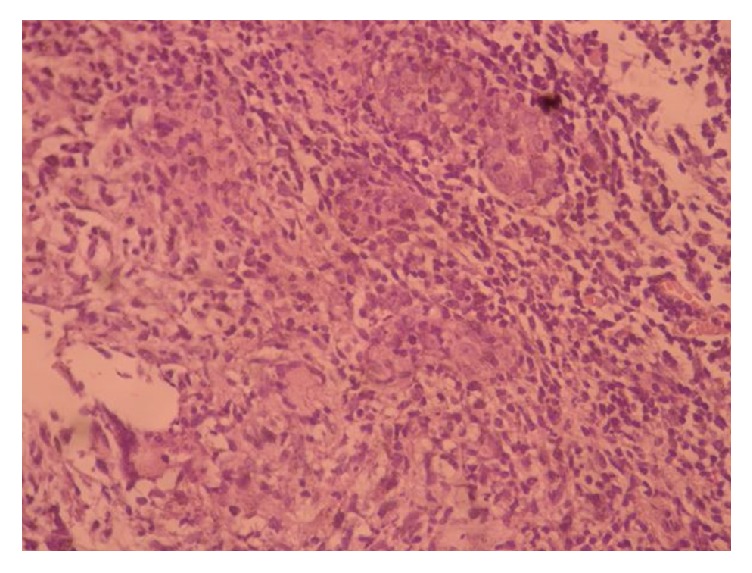
IGM histopathology H-E ×40.

**Figure 4:**
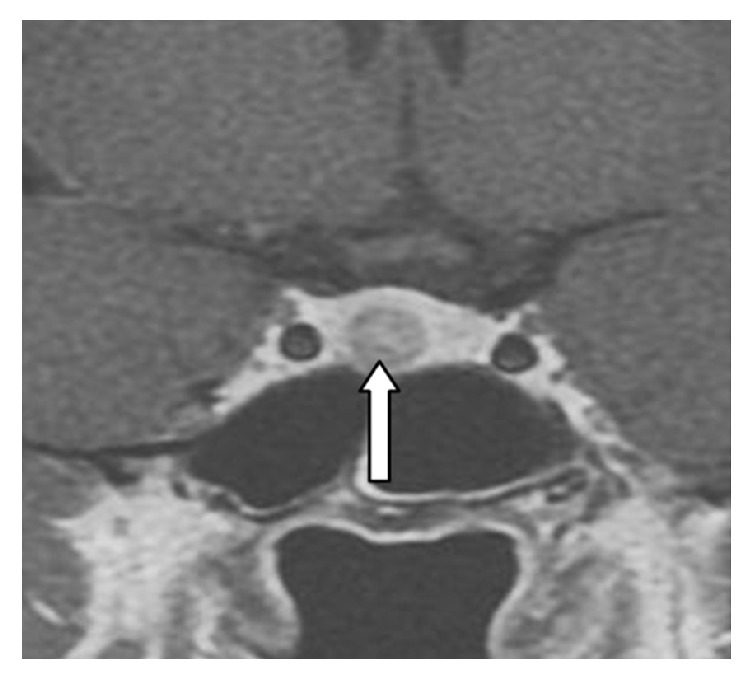
MRI of the pituitary microadenoma.

A thirty-nine-year-old single patient with no children and no history of oral contraceptive use was admitted to our clinic with complaints of redness, pain, and swelling in her left breast (Figure 1). She had no additional illness or complaints (BMI: 33.3). She was a tobacco user (5–10 units/day), within the normal weight category, and had three gravidity. Breast ultrasound revealed irregular limited solid heterogeneous hypoechoic mass lesions suspicious for malignancy; the largest one was 16 mm in diameter. There were no lymph nodes in the left axilla. The mass was categorized BIRADS-4 in breast ultrasonography. Breast MRI revealed heterogeneous enhancement with 3.5 × 5 cm of inflammatory area at the left breast upper outer quadrant. Biopsy was recommended for differential diagnosis of inflammatory breast cancer (Figure 2). Serum C reactive protein (CRP) was high (12.4 mg/l), sedimentation rate was high (37 mm/h), and CA 125 and CA 15-3 levels were normal. Gram (+) cocci were observed in the breast abscess stain; however, abscess culture results were negative. IGM was diagnosed with core biopsy examination (Figure 3). The breast abscess was drained and steroids were given for two months (Prednol 4 mg/day/oral and 0.1% betamethasone pomade) and empiric antibiotics (cefuroxime axetil 500 mg tablets 2 × 1) were given during treatment for ten days. After two months of treatment, there was no improvement. Therefore, body serum hormone profiles were examined. Growth hormone, insulin-like growth factor, thyroid stimulating hormone, estradiol, luteinizing hormone, and follicle-stimulating hormone were normal. However, serum prolactin was elevated (351 ng/ml). Pituitary MRI revealed a 7 × 4 mm sized microadenoma causing pituitary prolactinoma (Figure 4). In order to treat hyperprolactinemia, prolactin inhibitor (Cabergoline) was given to the patient. Cabergoline was started at 1 mg per week; then it increased for six weeks. After prolactin levels returned to normal, it was reduced. Cabergoline treatment was continued for two years.

Prolactin levels returned to normal and there was resolution of IGM after 4 months. Follow-up included monitoring of CRP levels. No recurrences were observed during a four-year follow-up period.

## 3. Discussion

In 1972, Kessler and Wolloch first defined granulomatous mastitis (GM) [1, 2]. GM can be idiopathic (primary) and specific (secondary) [2, 3]. Secondary GM involves caseation necrosis and emerges with a variety of infectious conditions such as vasculitis, sarcoidosis, tuberculosis, actinomycosis, and blastomycosis filariasis [2, 3]. IGM is detected in less than 1% of breast biopsies performed in women [1, 3]. It is also called idiopathic granulomatous lobulitis or idiopathic granulomatous lobular mastitis. It is often seen in women between the second and fourth decade of life. It is rarely detected in men [1]. This case report involved a 39-year-old woman.

The etiology of IGM is not fully understood, but autoimmune and hormonal disorders are often discussed as causes [2, 3]. IGM etiology, on the basis of inflammation and autoimmune effect, is reported to be in various pathological gene disorders [4]. IGM may appear together with autoimmune disorders such as Sjögren's syndrome, erythema nodosum, and arthritis [2, 4]. It is suggested that IGM can develop when T-lymphocytes mediated autoimmune reaction occurs against lobular epithelial cells and gastric secretions [2, 3]. Also, a close relationship between IGM and hormonal conditions such as pregnancy, lactation, use of oral contraceptives, and hyperprolactinemia has been demonstrated. In addition IGM is associated with *α*1-antitrypsin deficiency, diabetes, breast trauma, obesity, race, smoking, and infectious agents. IGM is more prevalent in the Mediterranean Region and in Asia [2]. This case report involved a tobacco user with normal weight and no history of oral contraceptive use.

A close association between IGM and hyperprolactinemia has been reported [2, 5]. Also, according to multiple reports, prolactin levels are important in recurrent cases [2, 4]. Hyperprolactinemia is mostly seen due to intense physiological conditions drugs such as phenothiazine, metoclopramide, risperidone, and pituitary adenomas [2, 5]. Researchers have demonstrated a relationship between IGM and pituitary adenoma that causes hyperprolactinemia [6, 7]. Prolactin plays a role in the inflammatory pathogenesis of the breast [7, 8]. Prolactin has a very important place in the proliferation and differentiation of normal breast epithelial tissue and in stimulating lactation after pregnancy [8]. Prolactin levels and/or increased expression of prolactin is thought to play a role in breast fibrocystic changes, ductal ectasia, benign breast lesions, and IGM and even in the development of breast carcinoma [8]. It has been reported that prolactin antagonist therapies cure IGM successfully when used in the treatment of hyperprolactinemia [5, 7]. In the case presented here, a pituitary adenoma causing hyperprolactinemia was found.

IGM presents with complaints such as painful breast mass, redness, abscess, and fistula and sometimes mimics cancer because of withdrawal of the nipple and peau d'orange appearance. It is rarely accompanied with axillary lymphadenopathy [3]. Usually, there is unilateral breast involvement; patients with bilateral breast involvement quarter are seen in [3, 4]. Hypoechoic tubular structures and nodules are seen in breast ultrasound; nodular opacities and focal asymmetry are seen in mammography. MRI findings are often nonspecific. Radiological results also mimic inflammatory breast cancer [1, 3].

Generally, in the absence of secondary infection, there is no growth in abscess culture [2, 3].

Gram stain and cultures should be performed in addition to PAS (periodic acid shift) staining for fungi and Ziehl-Neelsen stain for tuberculosis [3, 9]. IGM is diagnosed by the exclusion of other specific diseases that cause GM. A definite diagnosis is made by cytologic examination of fine needle and histopathological examination of core or excisional biopsies. Noncaseating granuloma in the lobular areas, giant cells, chronic inflammation, and microabscesses are seen in biopsy [9]. We applied Tru-cut biopsy in our patient and found similar clinical and radiological features to breast carcinoma.

Corticosteroids are the most common drugs administered for the treatment of IGM [3]. Other drugs used include anti-inflammatory drugs and immunosuppressive agents such as colchicine, methotrexate, or azathioprine [9]. Wide local excision or mastectomy can be applied in cases of medical treatment resistance, recurrent abscess, or fistula [10].

Recurrence rates can rise up to 50%. Follow-up recommendations include 3–6-month intervals for the first 2 years [9, 10]. In this case report, the breast abscess was drained and steroids, antibiotics, and prolactin inhibitor therapy was given to the patient. No recurrence was observed at four-year follow-up.

## 4. Conclusion

IGM is a disease associated with autoimmunity and hormonal disorders. When investigating the etiology of IGM, prolactin levels should always be checked. Prolactin elevation should be monitored particularly closely and continuously and for a prolonged period of time in severe cases. Prolactin elevation is known to increase immunity and inflammation. While investigating the causes of hyperprolactinemia, the primary focus should be the presence of a pituitary adenoma. Prolactin inhibitors will improve treatment success of IGM with reducing recurrence in cases with prolactin elevation.
